# Health system barriers to implementation of TB preventive strategies in South African primary care facilities

**DOI:** 10.1371/journal.pone.0212035

**Published:** 2019-02-14

**Authors:** Eva Van Ginderdeuren, Jean Bassett, Colleen Hanrahan, Lillian Mutunga, Annelies Van Rie

**Affiliations:** 1 Witkoppen Health and Welfare Centre, Johannesburg, South Africa; 2 Department of Epidemiology and Social Medicine (ESOC), University of Antwerp, Antwerp, Belgium; 3 Department of Epidemiology, Johns Hopkins Bloomberg School of Public Health, Baltimore, Maryland, United States; McGill University Faculty of Medicine, CANADA

## Abstract

**Background:**

Isoniazid preventive therapy (IPT) is a key component of TB/HIV control, but few countries achieve high IPT coverage.

**Methods:**

Using a behavioural COM-B design approach, the intervention consisted of a training on IPT guidelines and tuberculin skin testing (TST), identification of the optimal IPT implementation strategy by the health care workers (HCWs) of 3 primary care clinics, and a 2-month mentoring period. Using routine register data, TST and IPT uptake was determined 3 months before and 5 months after the intervention. Records were reviewed to identify factors associated with IPT initiation and HCW fidelity to the guidelines. A survey among HCWs was conducted to determine barriers to IPT.

**Results:**

Two clinics implemented TST-guided IPT for all clients receiving HIV care, one clinic decided against use of TST. According to routine register data, the proportion of clients initiating IPT increased substantially at the clinic not opting for TST (6% vs 36%), but minimally (34% vs 37% and 0.7% vs 3%) in the two other clinics. TST uptake did not increase (0 vs 0% and 0.5%). In addition to poor IPT uptake, HCW fidelity to investigation for TB and timing of IPT initiation was poor, with only 68% of symptomatic patients investigated and IPT initiation delayed to a median of 374 days post-ART initiation. In multivariate analysis, pregnancy (aOR 18.62, 95% CI 6.99–53.46), recent HIV diagnosis (aOR 3.65, 95% CI 1.73–7.41), being on ART (aOR 9.44, 95% CI 3.05–36.17), and CD4 <500 cells/mm^3^ (aOR 2.19, 95% CI 1.22–4.18) were associated with IPT initiation. Time needed to perform a TST, motivating patients to return for TST reading, and low IPT patient awareness were the main barriers to IPT implementation.

**Conclusion:**

Despite using a behavioural intervention framework including training and participatory development of the clinic IPT strategy, HCW fidelity to the guidelines was poor, resulting in low TST coverage and low IPT uptake under primary care conditions. To achieve the benefits of IPT, health system level approaches including TST-free guidelines and sensitization are needed.

## Introduction

TB continues to be a major public health problem with 10 million new TB cases and 1.4 million deaths worldwide in 2015 [1]. Isoniazid preventive therapy (IPT) reduces the progression of latent *Mycobacterium tuberculosis* infection to TB disease in those at high risk [2, 3]. The Global Plan to STOP TB aims to initiate IPT in 90% of all people living with HIV and 90% of all children in contact with TB by 2025 [4].

South Africa carries one of the world’s highest TB/HIV burdens, with close to one in every 100 South Africans developing active TB disease each year, and over 60% of all TB cases occurring in people living with HIV. The continued high TB notification rate highlights that the South African TB control strategy, which is mainly focused on passive case finding, fails to control the TB epidemic in South Africa [1]. Scaling up TB preventive strategies among people living with HIV, including early initiation of antiretroviral treatment (ART) and high uptake of IPT, may be essential to reduce the TB incidence in South Africa. While 56% of people living with HIV in South Africa are initiated on ART, less than 10% start IPT [5, 6].

Recent qualitative work assessing barriers to IPT implementation from the point of view of South African HIV care providers has suggested that the primary barrier to IPT use was lack of knowledge and experience [7]. Other barriers reported in the literature were inaccuracy of TB screening tools to exclude active TB, fear of isoniazid resistance, isoniazid and tuberculin shortage, and lack of clear guidance [8, 9]. Moreover, the reintroduction of TST as well as the complex stratification by ART status and TST status in the national 2014 South African IPT guidelines for people living with HIV likely poses logistical and human resource challenges to IPT implementation in already overburdened primary health care clinics (Box 1) [10, 11].

**Box 1. Overview of the WHO and South African IPT policy for people living with HIV**

**Table:** 

	WHO* [10]	South Africa [11]
IPT eligibility	All people living with HIV with negative symptom-based TB screening	All people living with HIV with negative symptom-based TB screening
TST provision	Use of TST where feasible.	Use of TST where available.
IPT duration	• TST positive or unknown: at least 36 months (as proxy for lifelong IPT), irrespective of ART • TST negative: assessed on a case-by-case basis for their individual risk of TB exposure	• TST positive: 36 months • TST negative: 0 (pre-ART) and 12 months (ART) • TST not available: start IPT and do TST after 1 m • TST not available by 6 months (pre-ART) or 12 months (ART): stop IPT

*in resource constrained settings with high TB prevalence

Improving the implementation of an evidence-based practice such as IPT necessitates successful behaviour change at the patient, provider and health system levels. The COM-B framework identifies three main components of behavioural change (‘Capability’, ‘Opportunity’, and ‘Motivation’) which can be targeted by interventions [12]. We used the COM-B model to identify barriers to IPT implementation in our setting, and to design an intervention to improve IPT uptake in three primary care clinics. This intervention included training to enhance capability, a participatory design to create opportunities for IPT initiation, and mentored implementation and feedback to increase motivation. Following implementation, we evaluated IPT and TST uptake, determined health care worker (HCW) fidelity to the 2014 South African IPT guidelines, and identified remaining barriers for IPT and TST implementation as perceived by HCWs.

## Methods

### Study setting

The study was performed in three primary care health facilities serving the Diepsloot community in Northern Johannesburg from August 2015 to November 2016. Diepsloot is a poor, densely populated, urban informal settlement of approximately 12 km^2^ with an estimated population of 138,329 [13]. The clinics routinely provide HIV and TB care in line with the South African guidelines but, at time of the study, the 2014 IPT guidelines were not yet implemented. Two clinics are DOH-funded without any history of implementation research, one clinic receives NGO and DOH funding and has participated in implementation research on TB, HIV and antenatal care. The South African Department of Health (DOH) issued new IPT guidelines in December 2014, which stratify eligibility and duration of IPT by TST and ART status [11]. According to these guidelines, TST-positive individuals are IPT eligible regardless of ART status and should receive isoniazid for 36 months (Box 1). TST negative individuals are only IPT eligible if on ART and should receive 12 months of isoniazid. If the TST is not done within one month of IPT-initiation, isoniazid should be administered for 6 months in people not on ART or for 12 months in people on ART.

### Study design

We used a before-after design to evaluate the change in uptake of IPT and TST following an intervention that consisted of (1) training, (2) participatory identification of the optimal IPT implementation strategy for each clinic, and (3) mentored implementation of the chosen strategy, followed by routine implementation.

#### Training of clinic staff

At each clinic, a 4-hour training session was organized for all clinic staff working with TB and HIV patients, with separate sessions tailored for clinicians, auxiliary nurses, counsellors and community health workers. The training was given by a study clinician in English, the language used by DOH for all staff training. The training covered the evidence on IPT effectiveness in people living with HIV and an in-depth overview of the 2014 South African DOH IPT guidelines. The theoretical knowledge covered was then applied to practical case studies to assure in-depth apprehension among all HCWs. During the last interactive session, all HCWs were encouraged to express concerns related to the IPT clinic flow and propose solutions. Clinicians were trained in TST placement using PPD RT/23 (2UT/0.1ml) and all relevant clinic staff were trained in TST reading. TST training was based on the Center for Disease Control and Prevention (CDC) TST training video and the TST training guide of the New Jersey Medical School Global Tuberculosis Institute [14, 15]. After the training, performance of TST placement and TST reading was assessed by the study staff.

#### Participatory development of IPT implementation strategy at each clinic

The clinic flow and clinic environment were mapped and reviewed by the clinic manager and the clinician(s) in charge of TB and HIV care taking into account all concerns and solutions identified during the training. Using a participatory and iterative process, options for sustainable integration of IPT and its documentation were identified at each of the three clinics. Solutions to create a facilitating environment included a fast-track system for TST reading visits, full integration of IPT visits (except for TST reading visit) in the normal HIV care program, cooperation with local clinics for TST reading and documentation of IPT and TST results on hand-held patient cards. Options were discussed until a consensus on the optimal and feasible strategy at each of the three clinics was reached.

#### Mentored IPT implementation

To support IPT implementation and enhance treatment literacy, each clinic was given educational materials including patient TST cards, pamphlets and posters (developed by the South African DOH in English and Zulu, the two most commonly spoken languages in South Africa), and a poster with instructions for TST reading and placement (developed by CDC).

To assure that the IPT strategy selected by the clinic was being implemented as intended, a study staff member was present to assist with IPT-related activities and monitor the relevant clinics’ processes and their documentation during the first 2 months of implementation. Study staff support was withdrawn after two months. Quarterly feedback on IPT uptake was given to the clinic managers.

### Data collection

Data on IPT uptake and TST placement was collected using routine clinic registers in paper and/or electronic format. These registers collected information on IPT initiation in all people newly diagnosed with HIV (clinic 1 and 2) or in all clients receiving HIV care (clinic 3). The number of people initiated on IPT and number of TSTs placed was extracted from these registers for the three months prior to the intervention and 5 months after completion of the 2-month mentored IPT implementation.

To assess the quality of the data collected in routine registers, data on IPT uptake was also collected through review of pharmacy reports of isoniazid mono-therapy prescriptions. This validation was only performed at one of the three clinics as detailed pharmacy records were not available at the other two clinics.

To determine HCW fidelity to the guidelines and identify factors associated with IPT initiation, we reviewed the records (paper medical files and electronic laboratory databases) of all clients receiving HIV care at one of 8 *a priori* selected days distributed throughout the 5 months following the 2-month mentored IPT implementation. This record review was only performed at two clinics as medical files are kept by patients in the third clinic. We extracted information on age and gender, CD4 count, viral load, IPT initiation prior to the day of record review, presence of contra-indications for IPT (current TB treatment, active liver disease, peripheral neuropathy, excessive alcohol use, history of adverse reactions to isoniazid, history of treatment for multidrug resistant TB), presence of symptoms suggestive of active TB (cough, fever >2 weeks, weight loss, night sweats), and results of sputum Xpert MTB/RIF, *M*. *tuberculosis* culture, and chest X-ray. We also documented whether TST was placed at that visit (and results thereof), whether IPT was initiated, and if so, its intended duration.

To identify important barriers to the IPT program, a survey was done during the last two months of the observation period among all HCWs involved in IPT prescription using a structured questionnaire based on the COM-B model. The survey enquired about competency (‘Capability’), habitual attitudes (‘Motivation’), and hurdles related to the clinic flow (‘Opportunity’).

### Data analysis

To assess IPT and TST uptake before and after the intervention, the proportion of clients initiated on IPT were compared using either all clinic clients newly diagnosed with HIV or any clinic client presenting for HIV care as the denominator for the population of interest, depending on the data routinely collected by the clinic. Chi-square was used to compare proportions.

To identify patient-level factors associated with initiation of IPT among those eligible, we performed a logistic regression analysis. Variables with a p-value ≤0.20 in the bivariate analysis were included and a backward selection method was employed to identify those factors independently associated with IPT initiation. The goodness-of-fit of the stepwise logistic regression model was assessed using the Hosmer-Lemeshow test.

To assess implementation fidelity, a descriptive analysis was performed by comparing the decisions made by the HCWs to the ones expected when guidelines would have been followed accurately.

To evaluate barriers to IPT implementation, frequencies and proportions were used to summarize the responses to closed survey questions.

All analyses were performed in R.

### Ethics approval

Ethical approval was obtained from the Health Research Ethics Committee of the University of the Witwatersrand (M150782, M160910). All identifying patient details were removed at time of data collection, recorded data were anonymous and securely stored. Waiver of consent was given by the ethics committee for review of routine clinic registers (clinic 1) and retrospective record review (clinic 2); all participants at clinic 3 gave written permission for use of routine data for research purposes.

## Results

### Selection of implementation strategies

All three clinics opted to implement IPT for all clients receiving HIV care, but only two clinics opted to implement TST (Table 1). Two different TST implementation strategies were used: a single nurse placing all TSTs (antenatal care service of clinic 3) or all clinicians placing TSTs in their patients (clinic 1 and general adult service of clinic 3).

**Table 1:** IPT and TST uptake during a 3-month period before and 5 months after an intervention consisting of training and a 2-month mentored implementation.

Clinic	Average monthly number of new HIV diag-noses	IPT strategy pre-intervention		IPT strategy identified through participatory process		TST placements		Average monthly proportion of IPT initiations*	
		TST screening	IPT eligi-bility	TST screening	IPT eligi-bility	Before inter-vention	After inter-vention	Before inter-vention	After inter-vention
**1**	68	No	All PLWH emphasis on pregnant women	Yes	All PLWH	0%	0%	34%	37%
**2**	38	No	All PLWH emphasis on pregnant women	No	All PLWH	NA	NA	6%	36%
**3**	208	No	All PLWH emphasis on pregnant women	Yes	All PLWH	0%	0.5%	0.7%	3.0%

*Routine registers collected information on IPT initiation in all people newly diagnosed with HIV (clinic 1 and 2) or in all clients receiving HIV care (clinic 3).

### IPT uptake

IPT uptake increased in all clinics, but the increase was only substantial in one and statistically significant in two clinics. The proportion of clients newly diagnosed with HIV initiated on IPT increased from 6% before to 36% after the intervention in clinic 2 (p<0.01), the proportion of clients living with HIV initiated on IPT increased from 0.7% to 3% (p<0.01) in clinic 3 (Table 1), and the proportion of clients newly diagnosed with HIV initiated on IPT increased from 34% to 37% thereafter in clinic 1 (p = 0.57). At each of the clinics, there was large month-to-month variability in IPT uptake, which could, at least in part, be due to operational issues that clinics encountered during the post-intervention period such as isoniazid stock out, human resources shortages, and high staff turnover.

Review of the pharmacy records (in clinic 3) suggested potential underreporting of IPT in routine registers, with 5% of all 23,452 clients receiving HIV care being prescribed isoniazid monotherapy during 5 months post mentoring compared to 3% of these clients recorded in the registers to having been initiated on IPT during the same period (p<0.01).

### TST uptake

For patients receiving antenatal care, TSTs were–as intended- mostly (74%) done by 1 nurse. At the general adult HIV care and treatment clinic, all 22 clinicians were responsible for performing TST in their patients but only 12 placed one or more TSTs. The intervention failed to increase TST uptake, with no (0%) TST placement in the pre-intervention period and 0% (clinic 1) and 0.5% (clinic 3) of clients assessed by TST in the post-intervention period (Table 1). Of the 106 individuals in whom a TST was placed, most 89/106 (84%) returned for the reading between 48–72 hours and 21/89 (24%) were positive (≥ 5 mm induration).

### Healthcare worker fidelity to the guidelines

**Fig 1:**
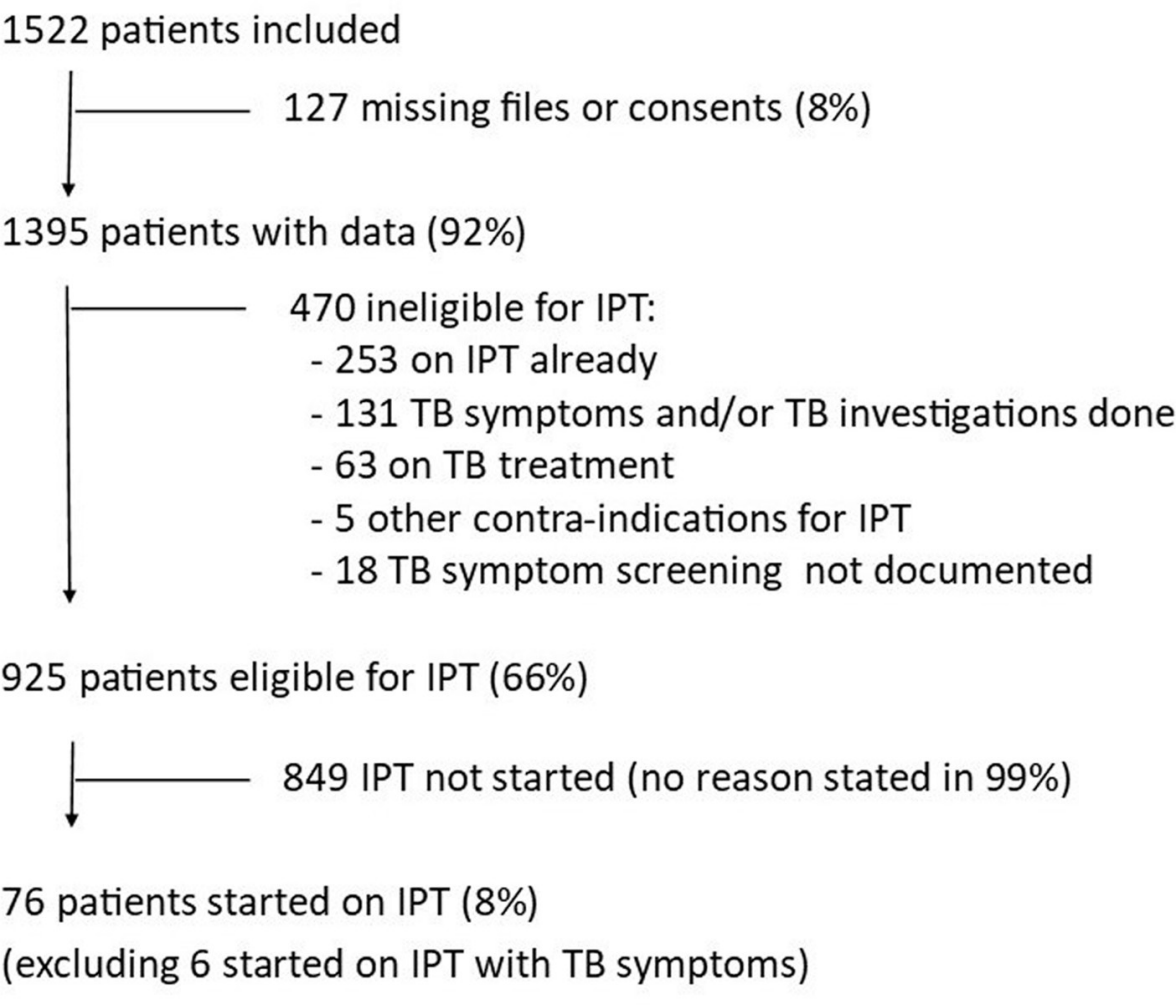
Flowchart of patients included in review.

Among the 1522 clients receiving HIV care during the *a priori* selected 8 days, 1395 (92%) had a medical file available for record review (Fig 1). Among these, IPT was not started in 63 (4.5%) patients because they were on TB treatment, in 253 (18%) because they were already on IPT, and in 5 patients because of a contra-indication for IPT (active liver disease n = 3, history of adverse reactions to isoniazid n = 1, or history of treatment for multidrug resistant TB n = 1). None of the records reviewed noted presence of peripheral neuropathy or excessive alcohol use as a contra-indication for IPT.

Among the remaining 1074 individuals, TB symptom screening was done in almost all (1056 or 98%) patients. Among the 1056 patients screened, 131 (12%) were classified to have presumptive TB. Overall, any investigation for active TB was performed in only 68% (89/131) of patients with presumptive TB, with large variability between clinics (22% in clinic 2 vs 71% in clinic 3, p<0.01). The most frequent initial diagnostic used was sputum Xpert MTB/RIF (in 80 of the 89 patients evaluated). The remaining 9 patients with presumptive TB were assessed by other investigations including sputum culture (n = 1), blood culture (n = 2), urinary LAM (n = 3), fine needle aspiration of lymph nodes (n = 3), chest X-ray (n = 3) and abdominal US (n = 1). Of the 68 Xpert MTB/RIF negative patients, 37 (54%) were assessed by culture, all of which was negative for *M*. *tuberculosis*. A TB diagnosis was made in 17 patients, based on sputum Xpert MTB/RIF (n = 10), AFB sputum (n = 1), chest X-ray (n = 1), blood culture (n = 1), and urinary LAM (n = 4). IPT was started in 6 patients with presumptive TB, five of whom had not been evaluated for TB and one patient whose chest X-ray and abdominal ultrasound were not suggestive of TB.

Among the 925 IPT eligible clinic clients (i.e. not on TB treatment, not receiving IPT and without TB symptoms), median age was 39 years (IQR 32–46) and two thirds (627 or 68%) were female, of which 24 (3.8%) were pregnant (Table 2). Most (805 or 87%) had been diagnosed with HIV more than 90 days prior to the record review visit and most (814 or 88%) were on ART. The most recent median CD4 count was 423 cells/mm^3^ (IQR 267–612) with a median time of 215 days (IQR 56–420) prior to the record review visit. Viral load suppression was present in half (52%) of the 736 clinic clients that had a viral load measurement available with a median time of 84 days (IQR 0–197) prior to the record review visit. A TST was performed at time of the record review visit in five of the 925 (0.5%) IPT eligible individuals. One TST was positive (14 mm), two were negative, and 2 patients did not return for TST reading. The TST result did not influence IPT eligibility as the two TST negative individuals were on ART. IPT was started in 76 of the 925 (8.2%) IPT eligible clinic clients, 63 of the 901 non-pregnant adults and 13 of the 24 pregnant women. Among non-pregnant adults, the median time from ART initiation to IPT initiation was 374 days (IQR 84–917) and the median CD4 count at IPT initiation was 374 cells/mm^3^. Among the 13 pregnant women started on IPT, two were not yet on ART at time of IPT initiation, six were on ART for more than one month, and five were on ART for less than 30 days. The intended IPT duration was specified in only 25 of 76 (33%) patients started on IPT but was according to the guidelines in all 25.

**Table 2:** Uni- and multivariate analysis of patient-level factors associated with IPT initiation among eligible patients.

		IPT started n = 76	IPT not started n = 849	OR (95% CI)	aOR (95% CI)
Gender	Female	52 (68%)	575 (68%)	1.03 (0.63–1.74)	
	Male	24 (32%)	274 (32%)		
Age in years (median (IQR))		34 (31–43.25)	39 (32–46)	0.96 (0.94–0.99)	
Timing of HIV diagnosis compared to IPT eligibility assessment	<90 days	19 (25%)	101 (12%)	2.47 (1.38–4.25)	3.65 (1.73–7.41)
	≥90 days	57 (75%)	748 (88%)		
ART status at time of IPT eligibility assessment	On ART	71 (93%)	743 (87%)	2.03 (0.88–5.87)	9.44 (3.05–36.17)
	Not on ART	5 (7%)	106 (12%)		
Pregnancy status	Pregnant	13 (17%)	11 (1%)	15.72 (6.77–37.20)	18.62 (6.99–53.46)
	Not pregnant	63 (83%)	838 (99%)		
HIV viral load*	Suppressed (<50 copies/ml)	28 (56%)	353 (51%)	1.20 (0.68–2.16)	
	Not suppressed	22 (44%)	333 (49%)		
Most recent CD4**	<500 cells/mm^3^	61 (80%)	509 (60%)	2.68 (1.54–4.97)	2.19 (1.22–4.18)
	≥500 cells/mm^3^	15 (20%)	336 (40%)		
Clinic	Clinic 2	12 (16%)	185 (22%)	0.67 (0.34–1.23)	
	Clinic 3	64 (84%)	664 (78%)		

*viral load missing in 26 patients who started IPT and 163 patients not started on IPT

**most recent CD4 count missing in 4 patients not started on IPT

Taken together, these data show that, except for screening for TB symptoms of all clinic clients living with HIV, HCW fidelity to the IPT guidelines was low, with only two-thirds (68%) of symptomatic patients investigated for TB, TST not implemented as intended (<1% coverage), low (8.2%) IPT initiation rate, and delayed initiation of IPT instead of as soon as “stable on ART” for non-pregnant adults or one month after ART initiation for pregnant women.

### Factors associated with IPT initiation

Age (OR 0.96, 95% CI 0.94–0.99), pregnancy (OR 15.72, 95% CI 6.77–37.20), recent (<90 days prior) HIV diagnosis (OR 2.47, 95% CI 1.38–4.25), ART status (OR 2.03, 95% CI 0.88–5.87) and CD4 <500 cells/mm^3^ (OR 2.68, 95% CI 1.54–4.97) were associated with IPT initiation in bivariate analysis. In multivariate analysis, pregnancy (aOR 18.62, 95% CI 6.99–53.46), recent HIV diagnosis (aOR 3.65, 95% CI 1.73–7.41), CD4 <500 cells/mm^3^ (aOR 2.19, 95% CI 1.22–4.18), and being on ART (aOR 9.44, 95% CI 3.05–36.17) remained independently associated with a higher odds of being initiated on IPT. Even among the patient groups where HCWs were more likely to initiate IPT, coverage of IPT was low: 54% of pregnant women, 16% of individuals with a recent HIV diagnosis, 11% of individuals with a CD4 count <500 cells/mm^3^, and 9% of people on ART.

### Barriers to IPT implementation as perceived by HCWs

The main barriers to IPT implementation reported by HCWs were low patient awareness of IPT, time needed to counsel patients on IPT, burden to document IPT-related activities, and concerns regarding exclusion of active TB (Table 3). Regarding TST, HCWs reported that the time needed to perform a TST and the difficulty to motivate patients to return for TST reading were important barriers to the use of TST.

**Table 3:** Self-reported barriers to IPT implementation among health care workers (HCWs).

	HCW (n = 25)
Systematic screening for IPT-eligibility is difficult to implement	1 (4%)
Current TB-screening is not sufficient to rule out TB	7 (28%)
Counseling patients about IPT is difficult/time consuming	7 (28%)
Patients not knowledgeable about IPT	18 (72%)
Documentation of IPT activities is difficult/time consuming	8 (32%)
Follow up of patients on IPT is difficult/time consuming^$^	4 (17%)
Motivating patients to adhere to IPT is difficult/time consuming	3 (12%)
Not enough training in IPT guidelines^¥^	0 (0%)
Not enough experience with prescribing IPT	0 (0%)
IPT not part of routine practice	1 (4%)
TST placement procedure is complex^¶^^,^^£^	2 (10%)
HCW not comfortable placing TST^¶^^,^^£^	0 (0%)
HCW has no time to perform TST^¶^^,^^§^	18 (86%)
TST is limiting factor in decision to place patient on IPT^¶^^,^^§^	0 (0%)
TST reading is time consuming^¶^	3 (14%)
Difficult to motivate patients to return for TST reading^¶^	15 (68%)

^$^1 HCW excluded as didn´t follow up patients on IPT

^¥^2 HCWs excluded as they started after training

^¶^denominator for TST only 2 clinics (n = 22), TST not performed in clinic 2 (n = 3)

^£^2 HCWs excluded as they referred for TST

^§^1 missing answer

## Discussion

Despite comprehensive training, participation by health care workers in the identification of the most sustainable IPT strategy for each clinic, post-training mentoring and feedback on performance, we observed poor fidelity to the IPT guidelines under routine primary care conditions resulting in low IPT uptake and almost non-existent use of TST. Pregnancy, recent HIV diagnosis, CD4 <500 cells/mm^3^, and being on ART were independently associated with higher IPT initiation, but even among those groups, IPT initiation remained low. The main barriers experienced among HCWs were concerns regarding exclusion of active TB, burden to document IPT-related activities, and operational issues with TST.

The low (8.2%) uptake of IPT was similar to the national IPT uptake estimates of approximately 10% [5] but far below the 80% target of the South African 2016 national strategic plan to scale up IPT implementation and far removed from the STOP TB target of 90% of people living with HIV initiating IPT [4, 16]. Our study highlighted potential inaccuracies in estimates of IPT uptake based on data recorded in routine public primary care clinic registers. This is important as register-based data on IPT uptake forms the basis for evaluation and strategic action by Departments of Health and WHO. In 2015, WHO changed the IPT denominator from all people living with HIV to only those newly enrolled in HIV care. WHO acknowledged that due to data quality issues, figures may not exclusively include number of people newly enrolled into HIV care, confounding interpretation and inter-country comparison of IPT uptake [1]. The use of different denominators across clinics (all clinic clients living with HIV or all patients newly enrolled in HIV care) may further complicate the collection of accurate summary estimates of IPT uptake at regional, provincial or national level. Finally, the observation that 18% of patients were on IPT at the time of file review but only 8.2% initiated IPT highlights the difficulty in interpretation of register-based estimates for IPT uptake. Register data provides a snapshot of IPT initiation at a particular clinic visit but fails to inform on the cumulative number of people living with HIV ever initiated on IPT. Reporting on cumulative indicators of “ever on IPT” may be required to evaluate progress towards the 90% IPT coverage target.

To date, few studies have assessed the operational feasibility, health systems aspects, HCW fidelity to IPT guidelines and resulting IPT coverage under real-life conditions. One study assessing IPT implementation in 49 South African health care facilities in 2010, when TST and ART status were not yet part of IPT eligibility assessment [17] found that IPT training was poor among clinic staff, IPT was only partly implemented in 71% of the facilities, and only half of eligible newly HIV diagnosed patients had been initiated on IPT. The TST requirement and the complex stratification by ART and TST status in the 2014 IPT guidelines may have contributed to the even lower (8.2%) IPT uptake observed in our study [11]. The finding that the only clinic to report a substantial and clinical relevant increase opted not to implement TST, supports this assumption. Two observational cohort studies in Médecins Sans Frontières (MSF) clinics in Kenya and Swaziland demonstrated that, in contrast to our finding of almost non-existent uptake of TST, the implementation of TST is feasible in an urban and rural resource-constrained settings [18]. The challenge in TST implementation we observed may have been due to greater human resource challenges in public sector clinics compared to MSF clinics. We could not compare our observation of low implementation fidelity to the 2014 IPT guidelines to findings in other studies as we could not identify any published reports. The low implementation fidelity resulted not only in poor uptake but also delayed IPT initiation, which results in missed opportunities to prevent TB among people living with HIV as the incidence of TB is the highest in individuals with low CD4 count and during the first months after ART initiation [19,20].

Recent qualitative work suggested that lack of knowledge and experience were important barriers to IPT implementation [7]. In our study, where HCWs were competently trained, we confirmed findings from other studies that low patient awareness of IPT and concern regarding exclusion of active TB posed barriers to IPT implementation [8, 9, 18]. Studies showed that increasing capability via knowledge transfer is not enough but needs to be complemented with supervisory mentored visits, staff motivation and a participatory environment [21]. To facilitate effective implementation of IPT, successful behaviour change of all 3 main B-COM components (‘Capability’, ‘Opportunity´, and ‘Motivation’) on patient, provider and system level will be mandatory. Our intervention showed that this may still not be sufficient and that additional approaches from a health system level are essential to create a facilitating environment that supports patient and care providers capability and motivation. Health system level approaches such as sensitization, political commitment, investment in human resources and continuous training, simplified guidelines and use of short regimens (such as one month rifapentine plus isoniazid [22]) may be needed to ultimately achieve high IPT coverage among people living with HIV.

Our study had multiple strengths, including the use of a behavioural framework for intervention design and evaluation, the participation of health care workers in the design of the IPT implementation strategy at each clinic, the mixed methods approach, rigorous file review, large sample size, and evaluation of three primary care clinics under routine conditions. Our study also suffered from limitations. First, all clinics serve the same community, limiting generalizability to other urban and rural areas. Second, patient refusal can contribute to low IPT uptake, but we lacked sufficient data to assess this. Third, the study was conducted before South Africa implemented ART for all, limiting generalizability of our findings to the current situation.

## Conclusion

The global call for TB elimination underpins the urgent need to scale-up TB preventive therapy. IPT implementation under routine primary care conditions is challenging due to numerous barriers related to concerns regarding exclusion of active TB, logistical and human resource challenges. To assure sustainable high uptake of IPT, simplified guidelines, HCW and patient sensitization on the benefit of IPT, and political commitment are needed to scale-up of IPT. Reporting on accurate standardized indicators on cumulative IPT uptake will be required to evaluate the success of the IPT program. Continued research towards shorter, better-tolerated regimens might be needed to ultimately achieve the 90% IPT coverage target.
